# Lipomics: A Potential Carrier for the Intravenous Delivery of Lipophilic and Hydrophilic Drugs

**DOI:** 10.3390/pharmaceutics14081651

**Published:** 2022-08-08

**Authors:** David Ramírez-Hernández, Carlos Juárez-Osornio, Vanessa Izquierdo-Sánchez, Pavel A. Figueroa-Rodríguez, Jorge Organista-Nava, Yazmín Gómez-Gómez, Luis Alberto Medina

**Affiliations:** 1 Facultad de Química, Universidad Nacional Autónoma de México, Mexico City 04510, Mexico; 2 Unidad de Investigación Biomédica en Cáncer INCan-UNAM, Instituto Nacional de Cancerología, Mexico City 14080, Mexico; 3 Facultad de Ciencias Químico Biológicas, Universidad Autónoma de Guerrero, Chilpancingo 39090, Guerrero, Mexico; 4 Instituto de Física, Universidad Nacional Autónoma de México, Mexico City 04510, Mexico

**Keywords:** lipomic, lipid-based carriers, lipophilic drugs, hydrophobic drugs, microSPECT/CT imaging, Taguchi’s design

## Abstract

In the present work, we propose the development of a novel carrier that does not need organic solvents for its preparation and with the potential for the intravenous delivery of lipophilic and hydrophilic drugs. Named lipomics, this is a mixed colloid of micelles incorporated within a liposome. This system was designed through ternary diagrams and characterized by physicochemical techniques to determine the particle size, zeta potential, shape, morphology, and stability properties. The lipomics were subjected to electron microscopy (SEM, TEM, and STEM) to evaluate their physical size and morphology. Finally, pharmacokinetic studies were performed by radiolabeling the lipomics with Technetium-99m chelated with BMEDA to evaluate the in vivo biodistribution through techniques of molecular imaging (microSPECT/CT) in rats. Radiolabeling efficiency was used to compare the encapsulation efficiency of the hydrophilic and lipophilic molecules in lipomics and liposomes. According to the results, lipomics are potentially carriers of lipophilic and hydrophilic drugs.

## 1. Introduction

The development of pharmaceutical carriers has been one of the essential objectives of nanomedicine. Nanocarriers provide more efficient and less toxic treatments with cytotoxic drugs, diagnostic imaging through radionuclides or contrast agents, and the monitoring of diseases [1,2]. One of the most used nanocarriers is lipid-based nanosystems, such as liposomes; these particles have relevant advantages as drug delivery systems, for example, drug protection against enzymatic degradation, low toxicity, increased permeability, biocompatibility, and low immunogenicity [2,3]. However, some of these advantages are limited because of the low encapsulation efficiency of drugs with poor aqueous solubility.

The development of nanocarriers to transport low-solubility drugs is relevant because many drug candidates or diagnostic agents are lipophilic with poor solubility in water [3]. The therapeutic applications of hydrophobic drugs are associated with minimal absorption and low bioavailability; in addition, the aggregation of these drugs on intravenous administration causes problems of embolism and local toxicity [4]. On the other hand, low solubility is also an intrinsic property of many lipophilic drugs, which helps the molecule or drug penetrate the cell membrane and achieve important intracellular targets. Different micelles-forming surfactants, colloidal dispersions with a particle size of 5 to 100 nm, have been used to overcome the low solubility of certain drugs. An essential property of micelles is their ability to increase the solubility and bioavailability of poorly soluble or insoluble drugs [2,5].

In the present study, we describe a new system named lipomics, potentially carrying both lipophilic and hydrophilic drugs. Lipomic are lipid nanostructures composed of a mixture of phospholipids, which under specific physicochemical conditions, form a nucleus of mixed micelles (phospholipid monomers and surfactants) surrounded by a bilayer of phospholipids (a liposome membrane), which can transport hydrophilic drugs in the aqueous space of the core and lipophilic drugs within the micelles [6].

In the manufacturing of lipomics, Taguchi’s experimental design was used to identify controllable parameters that minimize variations that could affect the method’s reproducibility. Pseudo-ternary phase diagrams were used to determine the proportion of the excipients needed to obtain the best lipomic formulation. Physicochemical techniques to determine particle size, zeta potential, morphology, and in vitro stability properties were implemented. For the in vivo biodistribution, pharmacokinetic studies of ^99m^Tc-lipomics were performed by plasma sampling quantification and molecular imaging with a microSPECT/CT system at different times. Radiolabeling efficiency was used to compare the encapsulation efficiency of hydrophilic and lipophilic molecules in lipomics and liposomes.

## 2. Results

### 2.1. Cloud Point Temperature (Cp) 

**Figure 1:**
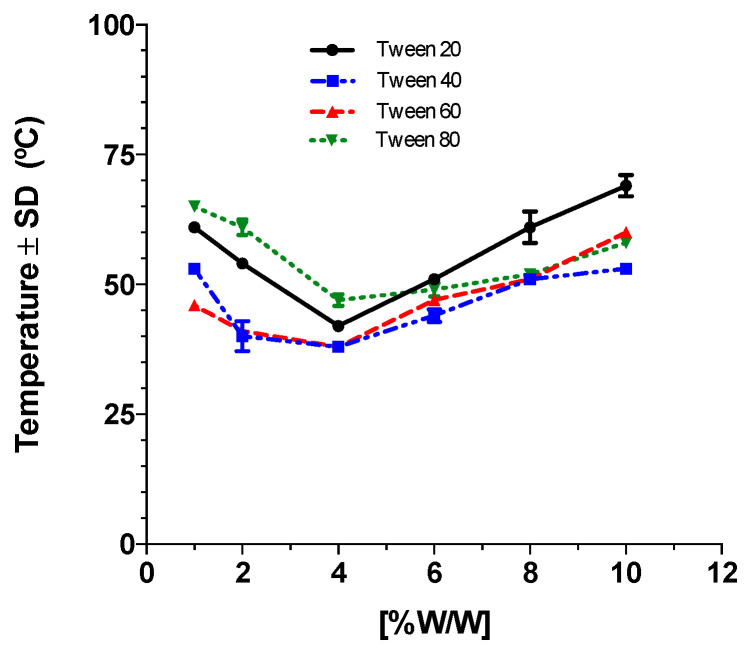
Determination of Cp for the different surfactants. Each point measurement was performed in triplicate.

Figure 1 presents the Cp for the different non-ionic surfactants used in the lipomic elaboration. The Cp measurement allowed us to obtain the temperature where the concentration of micelles was the highest at the interface, resulting in a stable micellar net that was apt to build the lipomics. Both the concentration and type of surfactants were evaluated; by keeping the concentration of ions in the medium constant, each surfactant had a different Cp. The analysis of Cp with the different surfactants determined the selection of Tween 80 for the elaboration of the lipomics, considering that a homogeneous surfactant solution with a high turbidity point (Cp) presents more stability. This temperature (47 °C) was also close to the transition temperature of the DSPC used in the formulation. The surfactants Tween 40 and 60 were discarded due to their low Cp in the concentration range needed to elaborate lipomics (0.5–6%*w*/*w*). Although Tween 20 has a high Cp (42 °C), it has the disadvantage of presenting a separation of phases in a short period of time after the lipomic preparation.

### 2.2. Taguchi’s Design

The orthogonal arrangement of Taguchi’s design resulted in sixteen different lipomic formulations prepared as a function of phospholipid, surfactant concentration, flow rate of isotonic saline infusion, and sonication time. Table 1 presents the sizes of the resultant lipomic structures for each formulation.

The results show that increments in the sonication time and flow rate reduces particle size; however, the variation in phospholipids and surfactant concentration is less conclusive. All formulations with diameters >1000 nm were discharged since particles larger than this size had low stability because of the separation of phases, coalescence between the suspended particles, and rapid elimination from the organism [7,8]. Only formulations 4, 8, and 12, with diameters <200 nm, were followed over a month by evaluating the particle size and zeta potential stability. The lipomic formulations with minor changes in these parameters were considered the most stable with a potential to be drug carriers.

### 2.3. Physicochemical Stability

**Figure 2:**
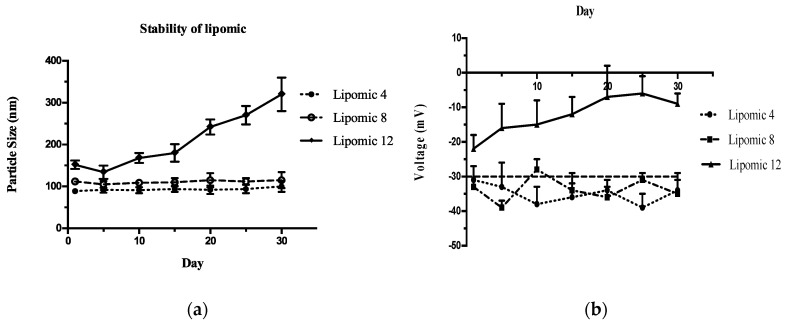
The evaluation of particle size and Z potential for one month, shown for formulations 4, 8, and 12. (**a**) The variations in particle size and (**b**) Z potential.

Figure 2 presents the results of particle size and zeta potential stability after a month, indicating that formulations 4 and 8 are the most stable. These results show that the electrostatic forces between the particles do not change with time since there is no increase in particle size or Z potential, avoiding agglomeration, fusion, coalescence, and the aggregation phase. These formulations were studied through pseudo-ternary phase diagrams to obtain information on the effect of excipients in lipomic size as their proportions were modified.

### 2.4. Ternary Diagrams

**Figure 3:**
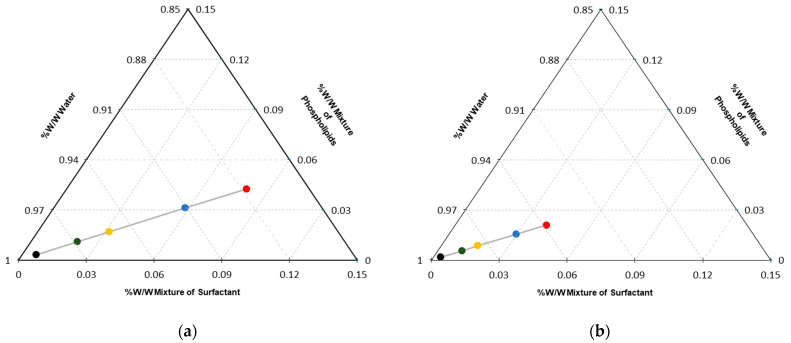
Pseudo-ternary phase diagrams: (**a**) the trajectories for formulation 4 and (**b**) formulation 8.

Figure 3 presents the pseudo-ternary phase diagrams for formulations 4 and 8. Each point in the trajectory represents a change in the percentage of the lipomic excipients expressed in %*w*/*w*. For both surfactants and phospholipids, when modifying their proportions in a mixture, they tended to change the lipomic characteristics. Monitoring each component was vital for the location of specific points in each trajectory. In this way, it was possible to find specific conditions in which the mixture of components produced a system with the desired physicochemical characteristics, in this case, the lipomic size.

The lipomic hydrodynamic size and population distribution for each point was calculated through the size histogram obtained from a Zetasizer Analyzer 90 PLUS/BI-MAS. Table 2 shows the results for both trajectories.

### 2.5. Electron Microscopies

**Figure 4:**
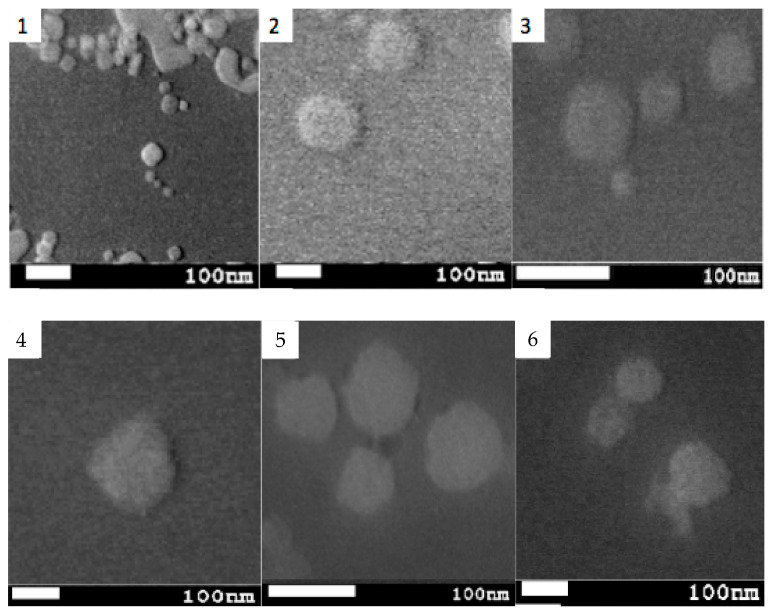
SEM images illustrating the lipomics’ size and morphology from (**top**) formulations 4.2 and (**bottom**) 8.2. Scale-bar is equivalent to 100 nm.

Figure 4 shows scanning microscopy pictures with morphological differences between both formulations. Lipomics from formulations 4.2 show some agglomeration of structures, (Picture 1) although the existence of isolated spherical particles is also observed (Pictures 2 and 3). Lipomics from formulation 8.2 mostly show spherical structures (Pictures 4–6). The average size measured in the images results in 86 ± 36 nm for lipomics in formulation 4.2 and 64 ± 19 nm in formulation 8.2, in agreement with the (hydrodynamic) size measured with the Zetasizer Analyzer (94 ± 10 and 73 ± 18 nm, respectively).

**Figure 5:**
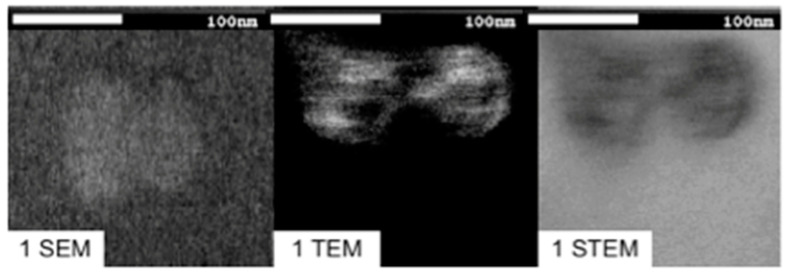
SEM, TEM, and STEM images of a sample of lipomics from formulation 8.2 loaded with uranyl acetate.

Figure 5 shows the microscopy images (SEM, TEM, and STEM) used to resolve the internal structure of lipomics from formulation 8.2; these were loaded with a hydrophilic contrast medium (uranyl acetate). The deformation was attributed to the break in lipomic osmolarity when adding uranyl acetate and the drying process to acquire the images. The TEM and STEM images show higher contrast regions, suggesting that the internal lipomic aqueous core is shared with micelles. No contrast medium (uranyl acetate) was used with SEM imaging.

### 2.6. Radiolabeling Results

Radiolabeling efficiency (RE) was used to compare the encapsulation efficiency of hydrophilic and lipophilic molecules in lipomics and liposomes. The BMEDA chelator used to carry ^99m^Tc through the lipidic membrane was transformed to its hydrophilic or lipophilic form through pH manipulation (pH < 6 or >7, respectively).

**Figure 6:**
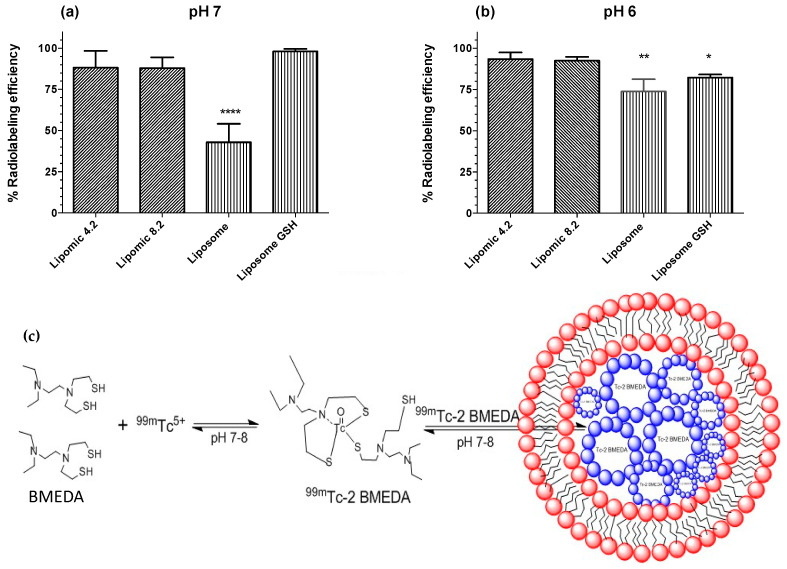
Radiolabeling efficiency of lipomics and liposomes as a function of pH to compare the encapsulation efficiency of lipophilic and hydrophilic molecules. (**a**) In lipophilic conditions (pH 7), (****) is statistically different from Lipomics 4.2 and 8.2, and liposome GSH. (**b**) In hydrophilic conditions (pH 6), (*) and (**) are statistically different from Lipomics 4.2 and 8.2. (**c**) The figure illustrates the encapsulation of lipophilic molecules (i.e., ^99m^Tc-2 BMEDA) in the micelles (blue structures in the figure) contained by lipomics.

The lipophilic conditions (pH > 7) and the osmotic gradient increase the passage of the ^99m^Tc-2BMEDA complex through the lipid membrane of lipomics and liposomes. For lipomics, the micelles at the core can catch and store large amounts of this lipophilic ^99m^Tc-complex, increasing the RE (Figure 6a,c). On the contrary, liposomes with their aqueous core repel the complex, pushing it out of the lipomic, reducing RE. In the case of liposomes with glucoheptonate (liposome GSH), GSH transforms the lipophilic complex into its hydrophilic form (^99m^Tc-BMEDA-H+), causing it to be trapped inside the core, resulting in a high RE. Statistically, there is no difference in the RE between lipomics and liposome GSH, making the radiolabeling of lipomics more practical by not requiring glucoheptonate.

In hydrophilic conditions (pH < 6), lipomics show a greater uptake (Figure 6b) of the ^99m^Tc-2BMEDA complex because of the osmotic gradient and the membrane’s flexibility with a low cholesterol concentration. Once inside, the hydrophilic ^99m^Tc complex stays in the aqueous core surrounded by the micelles, preventing the complex from quickly leaving the core. Similarly, the hydrophilic ^99m^Tc complex passes through the liposome membrane into the aqueous core; however, the complex can leave the core because of the osmotic gradient, reducing RE.

**Figure 7:**
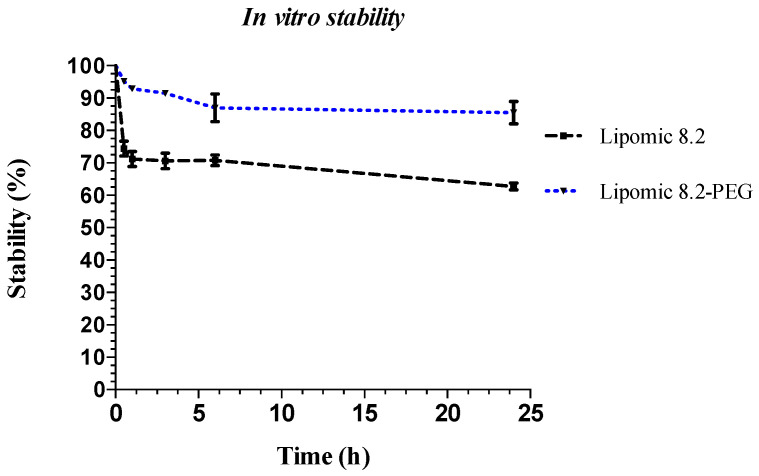
In vitro stability in serum of ^99m^Tc lipomics (formulation 8.2). The graph plots the mean value +/− standard deviation of three repetitions.

Figure 7 shows the in vitro stability in human serum of ^99m^Tc lipomics with and without PEG over 24 h at a temperature of 37 °C and constant agitation. The determination of stability in vitro helps to predict the behavior of lipomics in the body. The stability of any drug carrier is critical since, if the system is unstable, the carrier’s content would be released, causing toxic effects or a rapid elimination from the body. The results show that stability is more significant when the lipomic is PEG-coated, because the steric barrier that it produces reduces the interaction with plasma proteins and opsonization.

**Figure 8:**
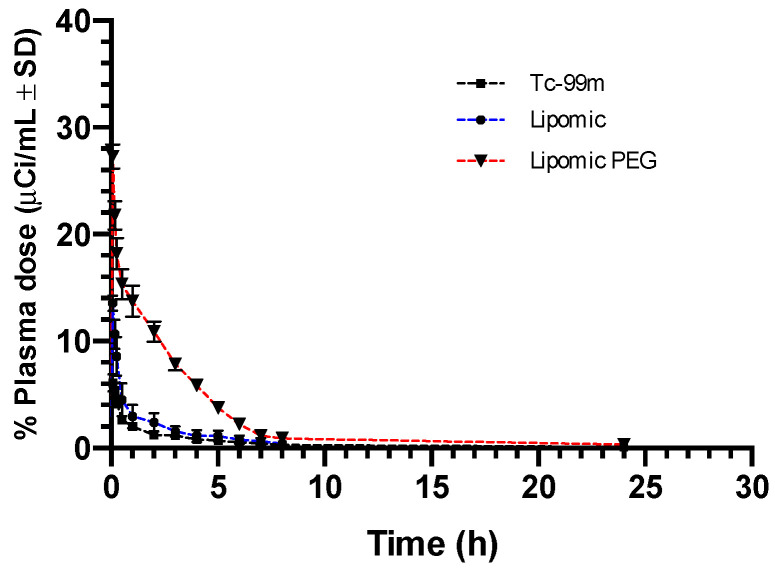
Plasma pharmacokinetics between ^99m^TcO^−^ and lipomics with and without PEG. The average of four repetitions ± standard deviation is reported.

In vivo pharmacokinetics of radiolabeling lipomics (formulation 8.2 with and without PEG) and “free” ^99m^TcO^−^ are presented in Figure 8, showing their differences. As compared with PEG lipomics, technetium is quickly removed from blood circulation.

Table 3 presents the pharmacokinetic parameters for radiolabeled lipomics and technetium, calculated using a two-compartment model. The pharmacokinetic parameters exhibit different behavior in the organism. In all three systems, it was observed that the half-life time in the central compartment (t_1/2_ α) was close to zero, which was corroborated by the half-life parameters in the peripheral compartment (t_1/2_ β) with a longer residence time. In addition, the lipomics’ interchange rate constant between compartments 1-2 (K_12_), with values significantly higher than those between compartments 2-1 (K_21_) and the elimination constant (K_10_), demonstrated that the flow of lipomics to the peripheral compartment was more significant than its return to blood circulation, indicating that the system was not eliminated, but was absorbed by the organs.

High ^99m^TcO^−^ and lipomics concentrations were observed in the peripheral compartment when comparing the volumes of distribution of compartments 1 and 2 (V_D1_ and V_D2_), where it was shown that V_D2_ was greater than that shown by V_D1_ with “free” ^99m^TcO^−^ being the most frequently captured by the peripheral compartment and in turn the one with the highest clearance (Cl). Clearance is a theoretical value that is associated with organ uptake.

Finally, the area under the curve (AUC_0-t_) represents the amount of drugs (i.e., ^99m^Tc) present in circulating blood over time; here, the PEG lipomics had the highest AUC_0-t_, which means that it remained in the blood longer; however, this does not mean that it remains in the body the longest.

### 2.7. In Vivo Imaging Biodistribution

The following SPECT/CT images show the representative biodistribution of “free” ^99m^TcO_4_^−^, ^99m^Tc lipomics, and ^99m^Tc-lipomics-PEG intravenously injected in healthy rats.

**Figure 9:**
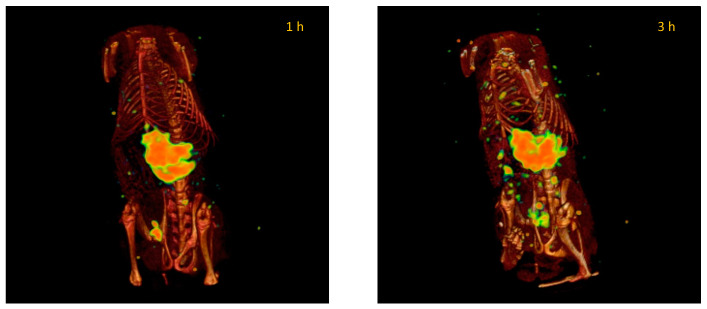
Representative SPECT/CT images of the ^99m^TcO_4_^−^ biodistribution in healthy rats at 1 and 3 h after injection. The accumulation mainly occurs in the stomach. Images are from the same rat.

For ^99m^TcO_4_^−^, the images were acquired at 1 and 3 h after administration because technetium is quickly removed from circulation after three hours, so imaging at 6 h was not considered. The metabolism of ^99m^TcO_4_^−^ is mainly linked to mucous membranes and glands, and the gastrointestinal tract is the main compartment for the metabolization and elimination of this radionuclide [9,10]. Its biodistribution (Figure 9) shows a majority uptake in the stomach, coinciding with what is reported in the literature [11].

**Figure 10:**
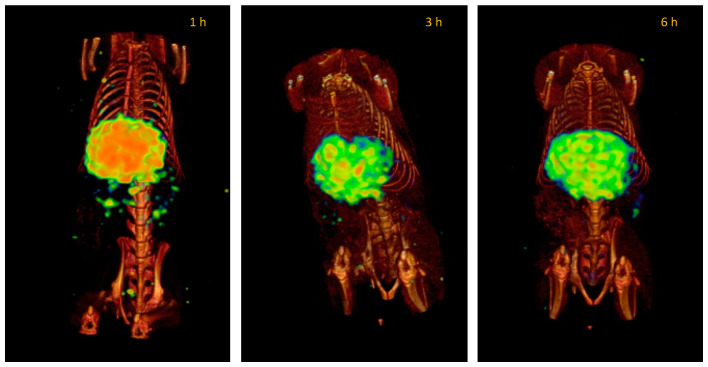
Representative SPECT/CT images of the lipomics’ biodistribution at 1, 3, and 6 h after injection in healthy rats. The accumulation mainly occurs in the liver. Images are from the same rat.

The lipomic systems that encapsulate ^99m^TcO_4_^−^ show a different biodistribution attributed to the phospholipid bilayer; this bilayer membrane prevents technetium’s direct interaction with its natural receptors delaying its elimination from the body. When lipomics are manufactured without PEG, this presents a rapid accumulation in the liver, a common pathway for phospholipid metabolism, staying there for the following 6 h (Figure 10).

**Figure 11:**
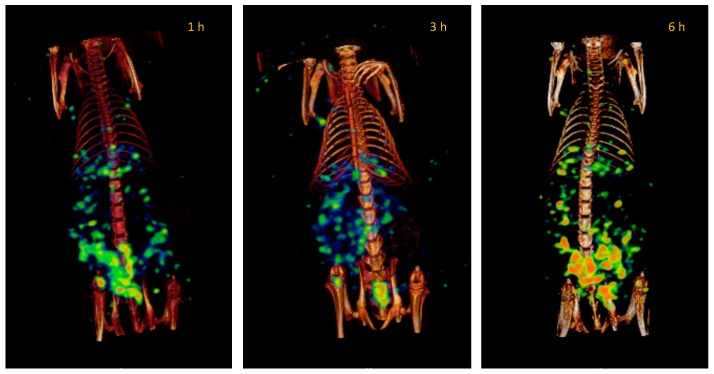
Representative SPECT/CT images of the PEG lipomics’ biodistribution at 1, 3, and 6 h after injection in healthy rats. Images are from the same rat.

In contrast, PEG lipomics have a steric barrier that can keep the lipomics in circulation before metabolization through the liver and spleen. The images show a biodistribution primarily concentrated in the intestines and liver (Figure 11).

**Figure 12:**
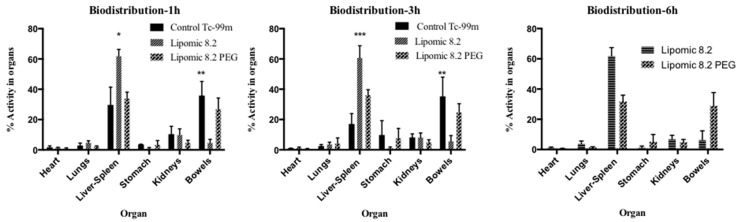
Uptake of ^99m^TcO_4_^−^, lipomics, and PEG lipomics as the percentage of activity present in each organ quantified from the images. The asterisks (*, **, ***) indicate statistical differences (*p* < 0.05) from the other groups.

**Figure 13:**
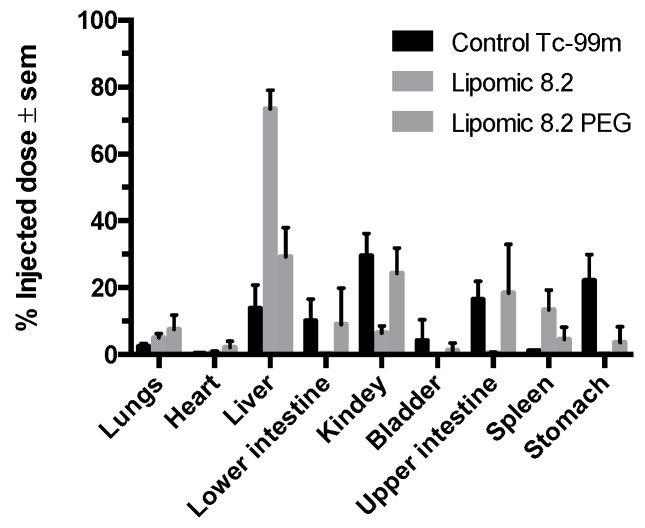
Biodistribution of ^99m^TcO_4_^−^, lipomics, and PEG lipomics as the percentage of injected dose (injected activity per organ weight) quantified after the animal sacrifice (n = 3–5), 3 h after injection.

Figure 12 shows the percentage of activity in organs for each formulation; it was quantified from the images at different acquisition times (1, 3, and 6 h). Figure 13 shows the ex vivo biodistribution in different organs 3 h after injection.

## 3. Discussion

From a previous work [6], it was suggested that using a unique combination of physicochemical parameters, based on pseudo-ternary phase diagrams, and experimental conditions, using Taguchi’s design, lipomics can be formed reproducibly.

The Taguchi method optimizes experimental parameters to minimize variation before optimizing the design to hit target values for output parameters. The method uses unique orthogonal arrays to study all the design factors with a minimum number of experiments [12]. Pseudo-ternary diagrams are widely used because they make it easier to understand the behavior of a system in equilibrium and determine the compositions of the phases and relative quantities of each of them [13]. In this work, the ternary diagrams allowed us to define the trajectory points that resulted in systems with the potential for use as carriers of lipophilic and hydrophilic drugs. Each diagram contains specific points placed from the concentrations of each of the components in the system, defining zones where the concentrations of the excipients tend to behave in a specific way. Then, it was possible to define the regions of interest where it was very likely to find systems with defined characteristics (i.e., size and morphology).

The surfactant’s cloud point (Cp) is an essential parameter in lipomic formation. When a surfactant reaches the Cp temperature, it forms a micellar network encapsulated by a lipid double bilayer of the phospholipids in the medium when the lipomic is assembled. The Cp mainly depends on surfactant concentration, its chemical structure (the extension of the carbon chain), and the concentration of ions in the medium. The surfactant concentration is directly related to the critical micellar concentration (CMC). By increasing the temperature, the CMC is increased, causing the formation of micelles. The non-ionic surfactants used in this work belonged to the family of ethoxylated surfactants; when the ethylene oxide units (hydrophilic portion) were increased and the carbon chain remained constant, the Cp increased. In contrast, by increasing the length of the carbon chain, the Cp decreased [14]. In this study, both concentrations and types of surfactants were modified, keeping the concentration of ions in the medium constant. Our analysis of Cp with the different surfactants determined the selection of Tween 80 for the elaboration of lipomics, considering that a homogeneous solution of surfactant with a high cloud point presents better stability.

Colloidal systems, such as liposomes and lipomics, are considered thermodynamically unstable. The physical stability of these systems depends on several factors: temperature, stress during transport inside the organism, compatibility of excipients, particle size, and the Zeta potential of the system [15]. A colloidal system ceases to be considered stable when the separation of its phases is irreversible; the most stable systems are those where the forces of repulsion predominate among the suspended particles, thus avoiding the coalescence and flocculation of the particles [16,17]. The physicochemical evaluation studies implemented in this work indicated that formulations 8 and 4 showed the best stability for 30 days (Figure 2). It was considered that the electrostatic forces between the particles did not suffer from alterations because in none of the systems was an increase in particle size greater than 150 nm observed, and the Z potential was maintained at around −30 mV. Therefore, the particles’ agglomeration, fusion, coalescence, and precipitation were avoided.

The shape and size of nanoparticles greatly influence their biodistribution and elimination from the organism [18]. Using SEM microscopy, it was possible to obtain images that allowed for the evaluation of these properties [19]. Spherical geometry and particle sizes around 100–200 nm are qualities that allow low detection rates by the immune system and, theoretically, a prolonged circulation time in the body [20]. With TEM/STEM microscopy, we sought to evaluate the internal structure of lipomics. Including a hydrophilic contrast medium in lipomics, such as uranyl acetate, highlights the hydrophilic regions inside. The electrons impinging on the uranyl acetate molecule are stopped, translating the image into regions with greater contrast and hydrophilic characteristics [21].

A second stage in this work was the radiolabeling process, reducing ^99m^TcO_4_^−^ with SnCl_2_, which formed a stable octahedral complex [22]. The ^99m^Tc–2BMEDA complex has a complexation pattern “SNS/S” (Figure 6c) that is the nucleus of technetium attached on the one hand to two atoms of sulfur and one of nitrogen, and is also attached to another atom of sulfur belonging to a second molecule of BMEDA [23]. The result of the process produces a complex with a hydrophobic or hydrophilic character dependent on pH [24]. Radiolabeling procedures also allowed us to obtain information about the structure of lipomics, considering that a hydrophilic chelator will find it more challenging to cross the lipid bilayer without the help of a concentration gradient or carrier. When performing the experiments with a concentration gradient of glucoheptonate, it was observed that the radiolabeling efficiency of hydrophilic molecules for liposomes was above 85% (Figure 6a), observing a better efficiency in lipomics. This result suggests the presence of hydrophilic compartments within the lipomics. When the same chelator was modified to a pH 7–8, it acquired lipophilic properties, facilitating its passage through the lipid bilayer. In these conditions, lipomics and liposomes did not show statistically significant differences, since both systems could carry more than 90% of the ^99m^Tc inside (Figure 6b). For lipomics, once the lipophilic complex of technetium entered through the lipid bilayer, the aqueous molecules inside drove the lipophilic molecules into the micelles, where they were trapped (Figure 6c). On the other hand, liposomes did not achieve an uptake greater than 50% in lipophilic conditions due to the repulsion of the lipophilic complex through its aqueous interior (Figure 6b). The results of the radiolabeling combined with the results of electronic microscopy suggest the existence of lipomic systems, also showing its hybrid nature, being able to “load” both hydrophilic and lipophilic compounds and being more efficient than liposomes by not requiring the use of glucoheptonate.

Solubility has a crucial role in the success of any drug candidate because low solubility not only causes problems for in vitro and in vivo assays, but also adds significant burdens to drug development. The delivery of hydrophobic chemotherapeutic drugs via the intravenous route remains a great challenge as these therapeutic molecules are associated with poor pharmacokinetics and bioavailability. In the present study, we obtained a nanocarrier-based biocompatible, non-toxic, and stable system, which can potentially address some of the problems associated with hydrophobic drug encapsulation. Pegylated lipomics as nanocarrier systems are promising for drug delivery; in addition to improving the pharmacokinetics of lipophilic drugs by internalizing them in the micelles core, the lipomics may allow specific a combination of drug delivery by inherent passive-targeting and active-targeting strategies.

Plasma pharmacokinetics and molecular imaging studies present an overview of the kinetic behavior of systems within the organism. When comparing the biodistribution of “free” ^99m^TcO^−^ with radiolabeled lipomics, with and without PEG, pharmacokinetic differences are evident (Figure 9, Figure 10 and Figure 11). By not presenting a protective coating, such as PEG, the differences in the circulation time of ^99m^TcO^−^ and ^99m^Tc lipomics are not statistically significant. Liposomes without this coating have shown circulation for approximately 90 min [25], matching our results (Figure 8, Table 3). The technetium, a free molecule smaller than lipomics, is quickly removed from circulation through the renal and glandular systems. On the other hand, pegylated lipomics accumulate in the liver and spleen, although a more heterogeneous biodistribution is observed in the intestines and stomach. Rapid metabolization in the liver and spleen may cause the release of ^99m^TcO- and, once free, it follows its normal metabolism pathway.

The potential of lipomics to carry both hydrophilic and lipophilic compounds needs to be evaluated with drugs commonly used in medical treatment, and cancer therapy, for example. The main limitation of anticancer drugs, such as taxanes (paclitaxel, docetaxel, and taxol), cisplatin, or gemcitabine is their lack of aqueous solubility, a magnified issue considering the need for its administration by intravenous infusions. Some solubilization strategies for these drugs include using surfactants and co-solvents that also result in adverse side effects [26]. The implemented procedures used here to elaborate lipomics offer a novel alternative that could minimize adverse effects; no solvents were used in the lipomic elaboration, and surfactants used to form micelles were encapsulated, avoiding its direct interaction that could induce side effects.

The quantitative analysis of biodistribution at 6 h (Figure 12 and Figure 13) indicated that non-pegylated lipomics accumulated almost exclusively in the liver, as it is the organ responsible for the metabolism of phospholipids. These lipomics could be used as carriers for drugs related to liver fibrosis, a distinct stage of chronic liver diseases that need novel treatments to block and reverse the underlying pathological process [27]. For example, silymarin and curcumin have promising effects for liver fibrosis; however, their poor bioavailability, water solubility, toxicity, and targeting efficiency limit their application [27,28]; their encapsulation in lipomics could help to reduce these limitations. Other drugs used for liver fibrosis treatment are under investigation, and nanocarriers, such as lipomics, offer a promising field of research to treat this disease. Notably, this carrier showed potential benefits similar to other carriers for poorly water-soluble drugs [26,29] that include improved pharmacokinetics and circulation half-life, passive targeting of the liver leading to improved treatments reducing systemic side effects, allowing the co-delivery of hydrophilic and lipophilic drug combinations in a single carrier, and the protection of drugs from quick degradation. As mentioned before, no solvents were used in its preparation, allowing us to minimize the toxic effects on the liver.

## 4. Materials and Methods

For the manufacturing of lipomics and liposomes, the following excipients were used: 1,2-Distearoyl-sn-Glycero-3-Phosphatidylcholine (DSPC) (Northern Lipids INC, Burnaby, BC, Canada), soy lecithin (Droguería Cosmopolitan, Ciudad de México, México), cholesterol (Chol) and N-(Carbonyl-Methoxypolyethylene glycol 2000)-Dystearoyl-Glycerophosphoethanolamine (DSPE-mPEG (2000) (T&T Scientific Corp, Knoxville, TN, USA), polyoxyethylenesorbitan monolaurate (Tween^®^ 20), polyoxyethylene sorbite monopalmitate (Tween^®^ 40), polyethylene glycol sorbitan monostearate (Tween^®^ 60) and polyoxyethylenesorbitan monoeleate (Tween^®^ 80), dextrose, glucohepptonic acid α−D-sodium salt (GSH), ammonium sulfate ((NH4)2SO4) (Sigma-Aldrich St. Louis, MO, USA), uranyl acetate (UO2(CH3COO)22H2O) (Specialty Chemistry of the Northwest, Sonora, Mexico), chloroform (CHCl3) (Reproquifin, Ecatepec de Morelos, Mexico), and methanol (CH3OH) (Honeywell Research Chemicals, Charlotte, NC, USA).

For the physicochemical evaluation, BI-ZR3 Zeta, a standard for potential (Brookhaven Instruments Corporation, USA); a particle-size reference standard made with NanospheresTM (Thermo Scientific, Waltham, MA, USA); potassium chloride (KCl); sodium chloride (NaCl); and ammonium ferrothiocyanate (NH4FeSCN) (Specialty Chemistry of the Northwest, Sonora, Mexico) were used.

In the radiolabeling, N,N-Bis(2-mercaptoethyl)-N′,N′-diethylenediamine (BMEDA), dimethylsulfoxide MB-grade (DMSO), tin chloride II (SnCl2) (Sigma-Aldrich St. Luis, MO, USA), nitrogen gas (PRAXAIR, Guildford, UK), hydrochloric acid (HCl) (Fermont, Monterrey, Mexico), acetic acid (CH3COOH) (Reproquifin, Ecatepec de Morelos, Mexico), and technetium pertechnetate (^99m^TcO^−^) (MIYMSA, CdMx, México) were used.

### 4.1. Taguchi’s Experimental Design 

Minitab software (Minitab Inc. Version 18) was used to design Taguchi’s experimental method. Four variables, (i) surfactant concentration; (ii) phospholipid; (iii) sonication time; and (iv) flow rate, with two levels of assessment (levels 1 and 2), were considered to measure the effect of particle size (Table 4). An orthogonal arrangement with sixteen possibilities was proposed (Table 5).

### 4.2. Determination of the Cloud Point (Cp) 

Four non-ionic surfactants (Tween^®^ 20, 40, 60, and 80) were used to determine the Cp; twelve solutions were elaborated at different concentrations in a weight-to-weight ratio (%*w*/*w*) in an isotonic saline solution (0.9% NaCl).

The Cp was determined with a homemade turbidimeter. This was created with an experimental setup consisting of a heating bracket (the grill) of one piece of copper and three 5 W resistors; a magnetic stirrer consisting of a motor with speed control by non-feedback PWM with a neodymium magnet; a vial holder; a thermocouple type K; an LED (the emitter) (5 W, 475 nm wavelength); a detector with a photodiode; and microcontroller to perform measurements and control the system. The equipment was validated with standard measurements before its use.

A vial with a non-ionic surfactant sample was placed on the stand and in contact with the grill. Then, agitation and temperature measurements were initiated. The LED (emitter) turned on and off with a square signal of 500 Hz. A trans-impedance amplifier supported the photodiode (receiver), and the voltage at its output was measured with the microcontroller. The first harmonic amplitude was obtained as a measure related to light intensity. The measurement of the light sensor at 30 °C was considered the baseline for determining the change in turbidity. The turbidity point was considered when there was a change of −3 dB from the initial measurement on the light sensor. The temperature at this point was recorded as the Cp.

### 4.3. Elaboration of Lipomics 

#### Empty Lipomics

The lipomics were prepared with a mixture of non-ionic surfactants (Tween 20, 40, 60, or 80) and phospholipids (DSPC, Chol, PEG-200) in a molar ratio of (DSPC/Tween/Chol/PEG-200) 60:30:5:5 mol. This mixture was placed in a ball flask, where it was homogenized using a magnetic stirring grill with moderate heating for about an hour; during this time, a volume of isotonic saline (NaCl 0.9%) was added by an infusion pump (baby-B). Subsequently, the ball flask was placed in a rotavapor (Buchi r-3, Switzerland) at 120 rpm for 1 h with sonication and at the temperature of the Cp of the non-ionic surfactant used. 

### 4.4. Elaboration of Liposomes 

#### 4.4.1. Empty Liposomes

In a sterile glass jar, the phospholipids (DSPC/Chol/PEG-200) were placed in a molar ratio of 60:35:5 mol, respectively. The phospholipid mixture was dissolved in 15 mL of a chloroform–methanol mixture in a 2:1 ratio. This solution was added, by slow drip, in 10 mL of sterile deionized water at 60 °C. The mixture was transferred to a ball flask and held for an hour of stirring at 120 rpm in a rotavapor (Buchi r-3, Flawil, Switzerland). Subsequently, the ball flask was placed inside a desiccator for 24 h to remove the remains of the solvents. 

#### 4.4.2. Manufacture of Liposomes Loaded with Glucoheptanate 

The same methodology previously described was used, but by replacing the 10 mL of deionized water with a solution of sodium glucoheptanate (GSH) with a concentration of 1 mg/mL, dissolved in a solution of ammonium sulfate ((NH4)2SO4) of 300 mM. At the end of the process, the liposomes were eluted in a PD-10 column (pH 7.4), previously balanced with a PBS solution. 

### 4.5. Physicochemical Evaluation 

#### 4.5.1. Ternary Diagrams 

For elaborating the pseudo-ternary phase diagrams, OriginPro software (OriginLab Corporation., Northampton, MA, USA) was used to visualize the variability in the proportion of components along each trajectory. 

#### 4.5.2. Particle Size and Z Potential

Dynamic Light-Scattering Spectrophotometry (DLS) determined the particle size and Z potential using Zetasizer Analyzer 90 PLUS/BI-MAS equipment (Brookhaven Instruments Corporation, Holtsville, NY, USA).

A total of 5 μL of polystyrene nanospheres were used as a reference standard (Duke Scientific, Palo Alto, CA, USA) dispersed in approximately 2 mL of KCl 10 mM, previously filtered (Millipore filter of 0.22 μm) to verify the proper functioning of the equipment. For sizing (hydrodynamic diameter), 10 μL samples of the lipomics diluted in approximately 2 mL of KCl 10 mM were used. 

The Zeta potential was determined using a palladium electrode with a clear four-sided acrylic cell. As a standard, BI-ZR3 Zeta was used in a previously filtered KCl 1 mM solution (Millipore filter of 0.22). The measurement was performed with 100 μL of sample diluted in 1.5 mL of KCl 1 mM. 

#### 4.5.3. Quantification of Phospholipids 

The quantification of the phospholipids was performed with Stewart’s method [30]. Briefly, 20 μL of lipomics or liposome samples were dried and reconstituted with chloroform (1mL); ammonium ferrothiocyanate (1 mL) was added and the mixture was shaken vigorously followed by centrifugation at 4 °C, 3000 rpm for 3 min (Centrifuge 5702R rotor #A-4–38, Eppendorf AG, Germany). The absorbance of the chloroform phase was measured with a UV-Vis DUO 530 spectrophotometer (Beckman Coulter, Brea, CA, USA) at a wavelength of 480 nm. A calibration curve (Abs vs. phospholipid concentration (mg/mL)), created with known concentrations of a solution of DSPC in chloroform, was used for quantification.

#### 4.5.4. Physicochemical Stability 

All lipomic solutions were stored in conical tubes (Thermo Fisher Scientific) at 4 °C; from these samples, an aliquot was obtained every 5 days, and the Zeta potential and particle size were measured to evaluate their stability for 30 days. 

#### 4.5.5. STEM, SEM, and TEM Microscopies 

Microscopy techniques were applied only to lipomic formulations with high stability over 30 days. Briefly, a dilution (1:100) was made of each sample, then a drop (~50 μL) was placed on a copper grid coated with 400-mesh carbon film and allowed to dry for 5 min. Then, the sample was stained with 2% uranyl acetate solution allowed to be absorbed by the lipomics. Subsequently, the samples were placed in an Ultra-High Resolution Scanning Electron Microscope JSM-7800F (Institute of Physics, UNAM). TEM and STEM techniques (electron beam at 30 kV) evaluated the morphology and size. No contrast medium (uranyl acetate) was used with SEM imaging.

### 4.6. Radiolabeling 

#### 4.6.1. Preparation of BMEDA Kits for Radiolabeling 

Two kits were prepared to implement radiolabeling. Kit 1 (to radiolabel lipomics) was prepared with 70 μL of BMEDA at a concentration of 0.5 mg/mL dissolved in dimethyl sulfoxide (DMSO) and 840 μL SnCl_2_ with a concentration of 10 mg/mL dissolved in HCl 0.1 M. Kit 2 (to radiolabel liposomes) contained the same components as Kit 1, plus 875 μL of GSH with a concentration of 50 mg/mL dissolved in 10% acetic acid *v*/*v*. The kits were vigorously stirred and degassed with nitrogen (N_2_) for 15 min. The final volume of each kit was divided into 11 Eppendorf tubes (Thermo Fisher Scientific). These tubes were frozen with liquid nitrogen and subjected to lyophilization (FreeZone 2.5, Labconco, Fort Scott, KS, USA). The freeze-dried samples (BMEDA kits) were stored under vacuum conditions in a desiccator until later use. 

#### 4.6.2. Radiolabeling of Liposomes and Lipomics 

BMEDA kits were resuspended in 5 mL of isotonic saline (NaCl 0.9%) and subsequently degassed for 15 min with N_2_. Then, HCl 1 M was added until a pH of 2 was obtained. From the above solution, an aliquot of 1 mL was obtained and placed in an Eppendorf tube, followed by adding 185 MBq (5 mCi) of technetium pertechnetate (^99m^TcO^−^); the mixture was vigorously stirred for 30 min. Later, the pH was modified by adding NaOH 1 M until reaching a value of 6 or 7, depending on the sample.

For radiolabeling, a volume of lipomics or liposomes, equivalent to 30 μmol of phospholipids (~0.5 mL), was added to ^99m^Tc-2BMEDA Kits 1 or 2, respectively, and incubated at 39 °C for 1 h at 300 rpm. Radiolabeling efficiency (RE) was estimated by instant thin-layer chromatography on silica-impregnated glass-fiber sheets (iTLC-SG) (Agilent Technologies, Santa Clara, CA, USA). Two microliters of RIC were spotted on 10 cm strips of the chromatography sheet paper; isotonic saline was used as the mobile phase. The strips were cut in half, and the radioactivity in each segment was measured using a well-type gamma counter (Ludlum 2200, Sweetwater, TX, USA). The *%RE* was calculated as:

*%RE* = 100 × [*B*1/(*T*1 *+ B*1)]

where *B*1 and *T*1 represent the radioactivity (cps) measured at the bottom and top iTLC-SG segments, respectively.

#### 4.6.3. In Vitro Radiolabeling Stability 

An aliquot of 20 μL of radiolabeled lipomics was obtained and added to 250 μL of human serum. Subsequently, the mixture was incubated in a dry bath at 37 °C with a constant stirring of 300 rpm (Multi-Therm, Thermo Fisher). Aliquots of 2.5 μL were obtained at 0, 0.5, 1, 3, 6, 12, and 24 h; %RE was measured as previously described. 

### 4.7. Pharmacokinetic Evaluation

#### 4.7.1. Animals 

Healthy male Wistar rats (6–8 weeks old) were obtained from the UNAM’s Medical School animal facility (Mexico City). The animals were kept in a pathogen-free environment and fed with autoclaved food and water ad libitum with photoperiod day/night 12/12, at a temperature of 22 °C and humidity of 40%. The procedures for the care and use of the animals were approved by the local Institutional Scientific and Ethics Committees at INCan (017/027/IBI)(CEI/1147/17), and all applicable governmental regulations (NOM-062-ZOO-1999, Ministry of Agriculture, Mexico City, Mexico) were followed. The guidelines from the Guide for the Care and Use of Laboratory Animals of the National Institute of Health (NIH, Bethesda, MD, USA) [31] were also followed. All efforts were made to minimize animal suffering and reduce the number of animals used in the experiments. 

#### 4.7.2. Blood Pharmacokinetics and Biodistribution Studies of Rats 

The rats were organized into three groups (n = 5). The control group was administered intravenously with 250 μCi of ^99m^TcO^−^, while experimental groups were intravenously injected with 300 μCi of ^99m^Tc-lipomics or ^99m^Tc-lipomics-PEG. Twenty microliters of blood drawn from the tail was collected at different times (min. to hours). The sample’s radioactivity was immediately measured on the well gamma counter (Ludlum 2200, Sweetwater, TX, USA). A graph of blood activity concentration (μCi/mL) against time was created, and the pharmacokinetic parameters were obtained from the calculation of a bicompartmental model. Some animals were sacrificed at 3 h, and the organs of interest were removed (spleen, heart, stomach, liver, small intestine, large intestine, lungs, kidneys, and bladder); these organs were weighed, and significant portions of the tissue were taken to quantify the activity in them. 

#### 4.7.3. Image Acquisition and Analysis

Imaging studies were performed at 1, 3, and 6 h after injection to observe the biodistribution of ^99m^TcO^−^, ^99m^Tc-lipomics, and ^99m^Tc-lipomics-PEG in the body. A microPET/SPECT/CT imaging system (Albira ARS, Bruker, Valencia, Spain) was used. During imaging, the rats were anesthetized using a mixture of oxygen/isoflurane at 3%. SPECT scans using a pinhole collimator were performed with 30 projections per detector at 60 s/projections. CT scans were performed with 600 projections (tube voltage 45 kV and 0.4 mA). SPECT images were reconstructed with Albira’s reconstruction software based on the ordered subsets expectation–maximization (OSEM) routine (three iterations). 

The percentage of activity at some specific time *t* (%A(*t*)) in the site of interest (organs) was calculated from the images as the ratio of the detected counts in the organ and the total number of counts detected in the whole body at baseline (A_0_), multiplied by 100 (%A(*t*) = A_organ_(*t*)/A_0_ × 100). A volume of interest (VOI) was drawn around the organ and the entire body on the images using PMODE software (PMOD Technologies LLC, Zurich, Switzerland) to determine the number of counts held. A vial with 5 μCi of ^99m^TcO^−^ was imaged and used to correlate counts in the VOIs with organ activity. Counts associated with the VOIs were corrected for radioactive decay with the image acquisition time.

### 4.8. Statistical Analysis

Values are reported as mean values ± standard deviation. Statistical analysis was performed using a one-way analysis of variance (ANOVA) on Prism 8 software (GraphPad Software, San Diego, CA, USA). The significance was determined at *p* < 0.05.

## 5. Conclusions

The poor aqueous solubility of drugs has been a serious limitation in clinical practice, mainly in the development of anti-cancer compounds that are regularly rejected because of their low solubility and high systemic toxicity. The higher dosing of many current chemo-drugs is also limited by the toxicity of high concentrations of surfactants/co-solvents used to solubilize the drugs. In this context, the lipomics elaborated in this work presented the potential to carry poorly water-soluble compounds offering additional advantages, such as the co-delivery of hydrophilic and lipophilic drugs, avoiding the use of solvents in its preparation. The system also showed an improved biodistribution and pharmacokinetics that will help to reduce systemic toxicity and protection of drugs from rapid degradation.

The implementation of the Taguchi method and ternary diagrams allowed for the development of a reproducible technique for the elaboration of lipomics. In addition, the physicochemical characterization of the lipomic demonstrated concrete evidence of the existence of the proposed structure. We intend to improve lipomics as a hybrid system to carry poorly water-soluble anticancer drugs and optimize the use of active-targeting strategies that will improve treatments.

## Figures and Tables

**Table 1:** Hydrodynamic diameter size and polydispersity index for lipomics from each formulation. Values represent the average and standard deviation of three independent experiments.

Formulation	Size (nm) ± SD	PI ± SD
Lipomic 1	658 ± 92	0.40 ± 0.05
Lipomic 2	326 ± 37	0.25 ± 0.03
Lipomic 3	395 ± 23	0.20 ± 0.01
Lipomic 4	89 ± 16	0.12 ± 0.01
Lipomic 5	2778 ± 36	0.46 ± 0.1
Lipomic 6	1170 ± 43	0.33 ± 0.09
Lipomic 7	2778 ± 56	0.14 ± 0.02
Lipomic 8	127 ± 15	0.19 ± 0.02
Lipomic 9	9990 ± 52	0.55 ± 0.015
Lipomic 10	1840 ± 67	0.24 ± 0.08
Lipomic 11	2779 ± 94	0.38 ± 0.07
Lipomic 12	158 ± 46	0.13 ± 0.01
Lipomic 13	11,495 ± 139	0.57 ± 0.02
Lipomic 14	936 ± 52	0.35 ± 0.02
Lipomic 15	1266 ± 40	0.20 ± 0.03
Lipomic 16	230 ± 41	0.16 ± 0.02

The polydispersity index (PI) measures the homogeneity of the sample regarding diameter. A value of 0.0 represents an entirely homogeneous sample, while a value of 1.0 represents an entirely heterogeneous sample.

**Table 2:** Hydrodynamic size distribution of lipomics obtained from the formulations elaborated with the different proportions (%*w*/*w*) of excipients.

			%Population Distribution	
Lipomic		Size Particle (nm) ± SD	50–200 nm	>200 nm
4.1	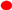	87 ± 31	89	11
4.2	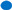	94 ± 10	100	0
4.3	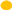	85 ± 31	69	31
4.4	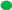	88 ± 39	88	12
4.5	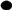	86 ± 19	88	12
8.1	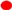	89 ± 49	69	31
8.2	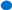	73 ± 18	97	3
8.3	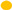	76 ± 24	84	16
8.4	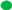	73 ± 27	66	34
8.5	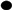	82 ± 64	80	20

Formulations 4.2 and 8.2, with size distribution close to 100% in population distribution (50–200 nm), were selected for electron microscopy analysis to evaluate the size and shape of the lipomics.

**Table 3:** Pharmacokinetic parameters of “free” ^99m^TcO^−^, ^99m^Tc lipomics, and ^99m^Tc-lipomics-PEG, calculated from a two-compartment model.

Parameter	^99m^TcO_4_^−^	Lipomic	Lipomic-PEG
t_1/2_ β (h)	2.2 ± 0.4	3.3 ± 0.1	3.5 ± 0.3
t_1/2_ α (h)	0.1 ± 0.05	0.5 ± 0.04	0.2 ± 0.03
K_21_ (h^−1^)	2.7 ± 0.4	0.7 ± 0.06	0.6 ± 0.2
K_12_ (h^−1^)	3.5 ± 0.7	6.7 ± 0.6	12.2 ± 2.3
K_10_ (h^−1^)	1.5 ± 0.3	0.9 ± 0.03	0.6 ± 0.6
V_D1_ (mL)	9.1 ± 1.7	6.5 ± 2.2	7.6 ± 1.3
V_D2_ (mL)	13.6 ± 2–5	16.1 ± 3.4	17.5 ± 2.6
Cl (mL/h)	18 ± 3.3	29.3 ± 1.8 *	31.8 ± 1.1
ABC 0-inf (µci·h)/mL	7.8 ± 0.6	10.3 ± 0.4	11.70 ± 0.8 *

The data correspond to the average (n = 5) ± standard deviation. (*) indicates a statistically significant difference from the other groups.

**Table 4:** Variables and levels of assessment.

Variable	Level 1	Level 2
Surfactant conc. (%*w*/*w*)	4.15	8.30
Phospholipid	DSPC	Lecithin
Sonication time (min)	30	60
Flow rate (mL/min)	0.33	0.16

**Table 5:** Taguchi’s experimental design.

Formulation	Phospholipid	Surfactant	Flow Rate	Sonication Time
Lipomic 1	1	1	1	1
Lipomic 2	1	1	1	2
Lipomic 3	1	1	2	1
Lipomic 4	1	1	2	2
Lipomic 5	1	2	1	1
Lipomic 6	1	2	1	2
Lipomic 7	1	2	2	1
Lipomic 8	1	2	2	2
Lipomic 9	2	1	1	1
Lipomic 10	2	1	1	2
Lipomic 11	2	1	2	1
Lipomic 12	2	1	2	2
Lipomic 13	2	2	1	1
Lipomic 14	2	2	1	2
Lipomic 15	2	2	2	1
Lipomic 16	2	2	2	2

## Data Availability

Data are contained within the article.
